# The role of LIN28B in tumor progression and metastasis in solid tumor entities

**DOI:** 10.32604/or.2023.028105

**Published:** 2023-04-10

**Authors:** TABEA GEWALT, KA-WON NOH, LYDIA MEDER

**Affiliations:** 1 Department I of Internal Medicine, Faculty of Medicine and University Hospital Cologne, University of Cologne, Cologne, Germany; 2 Center for Molecular Medicine Cologne, University of Cologne, Cologne, Germany; 3 Institute for Pathology, Faculty of Medicine and University Hospital Cologne, University of Cologne, Cologne, Germany; 4 Mildred Scheel School of Oncology, Faculty of Medicine and University Hospital Cologne, University of Cologne, Cologne, Germany

**Keywords:** Cancer, Metastasis, LIN28B, Let-7, Stemness, EMT, Resistance

## Abstract

LIN28B is an RNA-binding protein that targets a broad range of microRNAs and modulates their maturation and activity. Under normal conditions, LIN28B is exclusively expressed in embryogenic stem cells, blocking differentiation and promoting proliferation. In addition, it can play a role in epithelial-to-mesenchymal transition by repressing the biogenesis of let-7 microRNAs. In malignancies, LIN28B is frequently overexpressed, which is associated with increased tumor aggressiveness and metastatic properties. In this review, we discuss the molecular mechanisms of LIN28B in promoting tumor progression and metastasis in solid tumor entities and its potential use as a clinical therapeutic target and biomarker.

## Introduction

**Figure 1:**
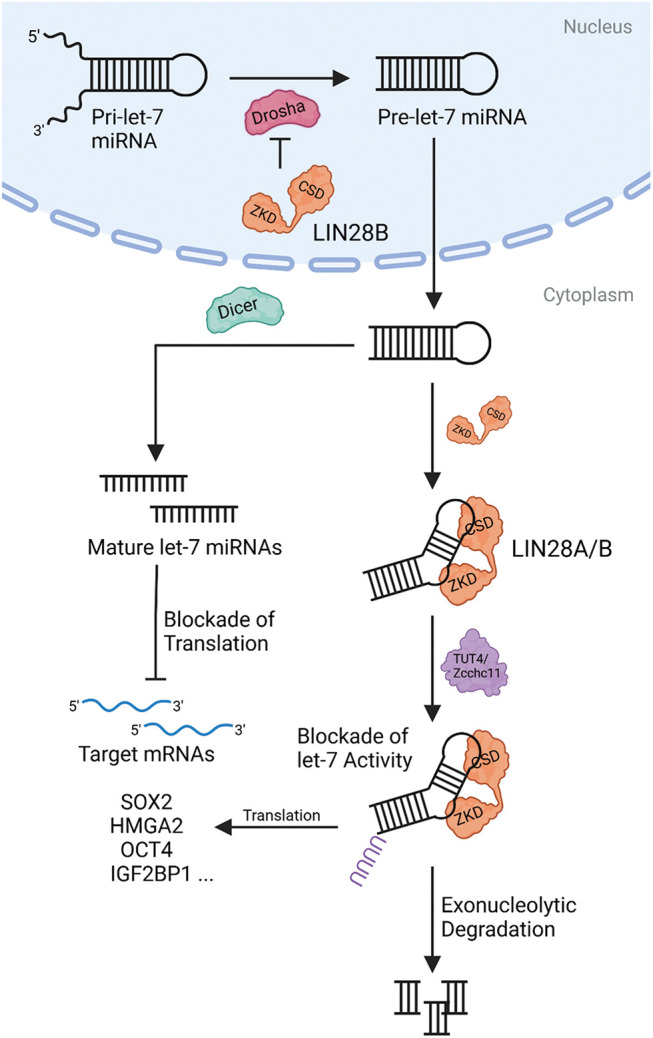
The molecular mechanism of LIN28B. LIN28B blocks Drosha from cleaving pri-let-7 miRNA into pre-let-7 miRNA in the nucleus. LIN28A/B can hinder the Dicer-dependent maturation of let-7 miRNA in the cytoplasm by binding let-7 miRNA loops via CSD and ZKD. Uridylation executed by the uridyl-transferase TUT4/Zcchc11 mediates let-7 degradation and translation of LIN28A/B targets. Created with BioRender.com.

LIN28B, one of the homologues of the Lin28 family, is an RNA-binding protein (RBP) and was first identified in hepatocellular carcinoma [1]. LIN28B represses the maturation and activity of microRNAs (miRNAs) and messenger RNAs (mRNAs) in embryonic tissues and undifferentiated cells, most notably of the let-7 miRNA family members, and prevents their accumulation in the early developmental stages. Later in differentiation, this correlation is reversed, resulting in low or absent LIN28B expression and indirect upregulation of LIN28B targets [2,3]. Generally, LIN28B consists of two functional domains: The N-terminal cold-shock domain (CSD) and the C-terminal zinc-knuckle domain (ZKD). These are responsible for the stepwise binding to pri- and pre-let-7 miRNAs, allowing for effective silencing of their biological activity and, eventually, degradation. The CSD initially binds to the terminal loop of the immature let-7 miRNAs, triggering let-7 remodeling and subsequent specific binding of the ZKD to a conserved GGAG motif on the let-7 miRNAs. As a result, the Dicer cleavage site of immature let-7 is blocked, as is its subsequent processing and cooperation into the RISC complex, where it normally executes its biological activity of silencing mRNAs with let-7 binding sites. Moreover, LIN28B binding recruits the terminal uridyl-transferase 4 (TUT4)/zinc finger CCHC-type 11 (Zcchc11), which promotes uridylation of the 3′-end of immature let-7 miRNAs and thus initiates inactivation and exonucleolytic degradation [4–6] (Fig. 1).

**Figure 2:**
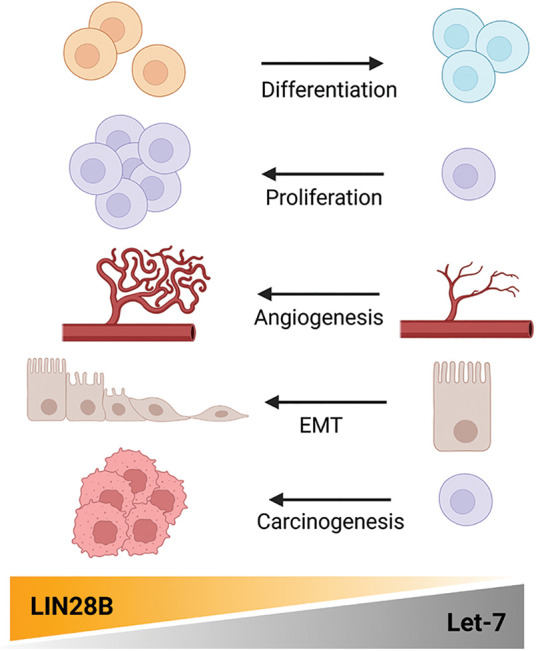
Pro-tumorigenic features driven by LIN28B. In tumorigenesis, LIN28B and let-7 act as antagonists, regulating cell differentiation, proliferation, tumor angiogenesis, epithelial-to-mesenchymal transition (EMT) and carcinogenesis itself. Created with BioRender.com.

Although LIN28B mutations have not been described as oncogenic, the presence of LIN28B mutations in the CSD domain in addition to the LIN28B missense mutations found on cBioportal suggests of some let-7 dependent pro-tumorigenic mechanisms [7,8]. Depending on the cell-cycle, LIN28B can be present in the nucleus, allowing for additional interaction with the immature pri-let-7 miRNAs. This can then lead to the interference of the Drosha complex, blocking their biogenesis [9]. Besides the binding to immature let-7 miRNA and the indirect gene regulation, LIN28B is able to regulate its target genes in a let-7 independent manner by binding to GGAGA motifs [4,10]. Different studies have shown that LIN28B also directly binds to specific mRNAs, such as SOX2, HMGA2, OCT4 or IGF2BP1, altering their translational efficiency and, ultimately, influencing several pathways involved in pluripotency, glucose metabolism or cell proliferation. Altogether, LIN28B activity promotes cell proliferation, epidermal-to-mesenchymal transition (EMT), angiogenesis, and the maintenance of a stem-cell like phenotype via the regulation of various miRNAs and mRNAs [9,11,12] (Fig. 2).

Above all, when LIN28B is aberrantly upregulated, the molecular effects of LIN28B activity becomes clear. It is frequently overexpressed in a variety of malignancies, including colon, breast, ovarian, and lung cancer. The oncogenic LIN28B protein, when overexpressed, plays a significant role in tumorigenesis, tumor progression and metastasis formation [13]. Furthermore, several publications reported that LIN28B is also involved in therapy resistance, leading to a poorer clinical outcome. Intrinsic LIN28B overexpression or induced LIN28B expression in tumor cells from various tumor entities can result in acquired resistance to chemo- or radiotherapy. Consequently, LIN28B can induce or reactivate EMT, proliferation, and survival features beneficial to tumor cells, reducing therapy efficacy and even causing tumor to relapse. Altogether, this makes LIN28B a potential therapeutic target as well as an intriguing biomarker molecule for early tumor grading [14–19].

This review provides a versatile insight into the crucial oncogenic roles of LIN28B in a broad range of solid tumor entities and its involvement in therapy resistance.

## LIN28B in Solid Tumor Entities

### Brain and nervous system

Brain and central nervous system (CNS) tumors are the most common types of cancer in children and the second most common in adolescents. The majority of brain and CNS tumors are malignant at this age, with an age-adjusted incidence of 3.55 per 100,000 [20]. Several studies provide evidence that LIN28B plays a crucial role in invasion and metastasis, particularly in malignant neuroblastoma.

In a study by Missios et al. [21], orthotopic mouse models injected under the left kidney capsule with bioluminescent LIN28B-expressing and LIN28B-deficient MYCN-amplified neuroblastoma cells were used to compare the metastatic burden on their livers. It was shown that *MYCN*-amplified neuroblastoma cells expressing LIN28B had a higher metastatic potential. Additionally, mice injected with cells expressing wildtype (WT) LIN28B showed a higher tumor burden relative to mice injected with cells containing mutant RNA-binding site of LIN28B [21]. This demonstrated that the RNA-binding ability of LIN28B plays a crucial role for the promotion of metastasis.

Furthermore, Chen et al. demonstrated that LIN28B is associated with metastasis and shortened overall survival *in vitro* and *in vivo*. Tumor sphere and wound migration assays of different neuroblastoma cell lines revealed increased proliferation and migration of control LIN28B-expressing neuroblastoma cells relative to LIN28B-knockdown neuroblastoma cells. In addition, immunocompromised mice injected intravenously with LIN28B-depleted cells had a lower tumor burden than mice injected with LIN28B-expressing cells. Moreover, they showed that PDZ binding kinase, a serine/threonine kinase that promotes the proliferation and self-renewal of neural stem cells, was positively correlated with LIN28B and MYCN expression in neuroblastoma cells [22]. Further to that, in 2015, Schnepp et al. discovered that LIN28B expression positively correlated with the expression of the oncogene *RAN*. Immunoprecipitation proved that the RAN mRNA was a direct target of LIN28B, thus resulting in its upregulation and to an enhanced neuroblastoma cell growth. The investigation of their downstream signaling finally disclosed that LIN28B indirectly promoted Aurora A kinase expression via upregulation of RAN expression and the downregulation of let-7, which is normally inhibited. Thus, LIN28B stimulated neuroblastoma cell proliferation by indirectly upregulating Aurora A kinase, which drives cell cycle progression [23]. Finally, two additional publications pronounce the crucial role of LIN28B in neuroblastoma initiation and progression [24,25].

To sum up, LIN28B is a master regulator of N-Myc and Aurora A kinase in neuroblastoma, and thus all three players drive their own feedback loops. Aurora-A builds a complex with N-Myc protecting it from proteasomal degradation [26], and it also forms a complex with hnRNPK to activate Myc transcription [27]. N-Myc regulates LIN28B expression levels indirectly by repressing miR-26a-5p and directly by binding of the LIN28B promoter [28].

Apart from the mechanism in neuroblastoma, it has been shown that LIN28B overexpression did not induce brain tumor formation originating from Nestin-positive neural precursors [29]. However, LIN28B is involved in tumorigenesis in glioblastoma originating from neural stem cells (NSC), NSC-derived astrocytes, and oligodendrocyte precursor cells [30–32]. The study of Yuan et al. published this year, explored the role and underlying mechanism of chemoresistance in glioblastoma. The initial qPCR experiments showed a significant overexpression of the long non-coding RNA LINC00520 in chemo-resistant glioblastoma cells. Silencing of LINC00520 markedly reduced cell viability, suppressed colony formation, and induced cell apoptosis. Further investigation revealed that the transcription factor Signal transducer and activator of transcription 3 (STAT3) bound to promoter regions of LINC00520 and that LINC00520 could also interact with LIN28B.

As a result, the authors argue that STAT3-mediated expression of LINC00520, which promotes the LIN28B expression, contribute to increased chemoresistance in glioblastoma through inhibited autophagy and reduced DNA damage [33].

Similarly to neuroblastoma, Aurora A kinase inhibition offers vulnerabilities in glioblastoma [34] and Ran was described to be strongly expressed in glioblastoma, enforcing patient-derived tumor spheres [35]. Thus, it is likely that LIN28B also acts in glioblastoma as a regulator of Aurora A kinase and Ran.

Taken together, these studies highlight the promoting effects of the RNA-binding protein LIN28B on tumor growth, tumor maintenance, and metastasis formation in brain cancers, particularly linked to Aurora kinases and N-Myc. Additionally, a role for LIN28B in chemoresistance is suggested. On the one hand, the presence of LIN28B expression will hardly change the clinical practice in treating neuroblastoma and glioblastoma patients with chemotherapy and combined interventions. On the other hand, a currently recruiting phase II trial (NCT04555837) combines the Aurora A kinase inhibitor Alisertib (MLN8237) in malignant solid neoplasm with the immune checkpoint blockade pembrolizumab, indicating a possible future for Aurora A kinase therapy combinations. LIN28B expression may benefit response rates in Aurora kinase inhibition and could be investigated as a stratification marker.

### Oral cavity

Oral cancer represents the collective tumor entities of the lip, the oral cavity, and the oropharynx, and is the 15^th^ leading cause of death worldwide [36,37]. Also in these tumor entities, RNA-binding proteins, including LIN28B, contribute to tumor aggressiveness, invasion, and metastasis [37]. Wu et al. showed that the expression of LIN28B was significantly higher in oral squamous cell carcinoma (OSCC) specimens than in adjacent normal tissue controls. A Kaplan-Meier analysis additionally revealed that OSCC patients with high LIN28B-expression had shorter overall and disease-free survival. In the same study, different *in vitro* assays, such as wound-healing assay, Boyden chamber invasion assay, and colony formation assay revealed that different OSCC cell lines with LIN28B-induced overexpression had higher migration and metastatic properties than controls [38]. A subsequent study confirmed this expression pattern of LIN28B via immunohistochemical staining in a chemical-induced OSCC animal model. High LIN28B expression was linked to epithelial dysplasia and invasive tumorous tissue, whereas LIN28B expression was nearly absent in normal epithelial tissue. Quantitative real-time PCR (qPCR) and Western Blot experiments validated these results in different OSCC cell lines compared to a control immortalized oral epithelial cell line. This LIN28B expression pattern was reinforced by histochemical scoring of LIN28B expression in a retrospective cohort of primary OSCC patients. Furthermore, high LIN28B expression was correlated with increased tumor size and clinical stage, indicating the association between LIN28B expression and tumor growth and aggressiveness [39].

In a study conducted by Lin et al. [40], LIN28B expression was found to be higher in tumorous tissues and even more in lymph node metastatic tissues when compared to adjacent normal tissue samples in a qPCR analysis of 20 matched pairs of tissues in OSCC patients. Higher levels of LIN28B expression were also correlated with advanced stages of OSCC. Different *in vitro* assays revealed that the knockdown of LIN28B abrogated the migration and invasion ability in two, the SG and FaDu LIN28B-expressing human OSCC cell lines. Also, the anchorage-independent growth and colony formation properties of these two cell lines were attenuated by LIN28B knockdown. These results further highlight that LIN28B plays a crucial role in the process of metastasis formation in OSCC [40]. Mechanistically, the study of Chien et al. elucidated how LIN28B regulated cancer stem cell features in OSCC [41].

LIN28B expression enhances Oct4 and Sox2 expression and stem-cell properties in a let-7 dependent manner. ARID3B and HMGA2 thus function as downstream effectors of LIN28B/Let-7 signaling. Interestingly, ARID3B is also described in neuroblastomas [42] and regulates together with N-Myc embryonic stem cell proliferation programs [43]. Consequently, it is likely that LIN28B plays a redundant role in tumorigenesis in different entities.

The studies collectively indicate that LIN28B overexpression is strongly linked to cancer stem-ness, tumor progression, metastasis formation, and poor prognosis in OSCC patients, indicating the clinical relevance of LIN28B in this tumor entity.

### Respiratory system

Generally, the respiratory tract can be divided into an upper and a lower part. The upper part consists of the nose and the larynx, whereas the lower part is represented by the lungs including the trachea, bronchi, bronchioles, alveolar ducts and alveoli [44]. Lung cancer is the leading cause of cancer-related deaths worldwide. Regrettably, most patients are only diagnosed at advanced stages of disease, and in spite of crucial developments in therapy options of late-stage lung cancer, survival is still poor. Thus, novel diagnostic biomarkers and therapeutic targets are urgently needed to improve the patients’ therapeutic outcome [45].

In lung cancer, LIN28B has been linked to radio-resistance [15], as well as increased proliferation and migration in association with miRNAs. Wang et al. showed that LIN28B knockdown in H1299 cells with high endogenous LIN28B expression reduced proliferation and migration compared to the control in *in vitro* assays of non-small cell lung cancer cell lines. Conversely, induced LIN28B expression of H460 tumor cells produced the opposite results. Furthermore, the proinflammatory Interleukin (IL)-1β significantly promoted proliferation and migration. Further investigation led to the identification that LIN28B was a molecular target of the miRNA-101. As a result, transfection of miRNA-101 in H1299 cells revealed a significantly decreased cell proliferation, colony formation, cell migration, and xenograft tumor growth. Altogether, they concluded that IL-1β indirectly upregulates LIN28B expression by suppressing miRNA-101 levels, thereby promoting LIN28B activity [46]. Another clinical analysis of lung adenocarcinoma depicted a positive correlation between LIN28B expression and advanced clinical stage, as well as an association between shortened overall survival. Clustered Regularly Interspaced Short Palindromic Repeats (CRISPR)/Cas9-mediated knockout of LIN28B in patient-derived lung adenocarcinoma cells dramatically attenuated proliferation, colony formation, migration, and invasion ability *in vitro*. In contrast, induced LIN28B overexpression promoted metastatic properties. An additional *in vivo* experiment showed that LIN28B expressing cells were more likely to form metastatic seeds on the liver surface when injected intravenously into nude mice. Mice injected with LIN28B-expressing cells also had a shorter survival rate than the mice injected with control cells. Finally, they could identify the long non-coding RNA LIN28B-AS1, located upstream from the *LIN28B* gene, positively regulated the biological activity of LIN28B [47].

The publication of Xiao et al. published in 2017 investigated the role of the miRNA-367-3p in non-small cell lung cancer (NSCLC) stem cells. Quantitative PCR analyses demonstrated a positive correlation between miRNA-367-3p and Wnt3 expression, however a negative correlation of miR-367-3p and miRNA let-7c in tumor tissues. A dual luciferase assay further revealed that miR-367-3p expression increased the promoter activity of LIN28B. This signifies that if miR-367-3p is upregulated, it indirectly increases LIN28B expression and Wnt signaling in tumor stem cells, sustaining their undifferentiated phenotype and self-renewal properties. These findings support the importance of LIN28B in tumor progression and the possible involvement of miRNAs [48]. Finally, the publications of Huang et al. and Zhou et al. provide additional insight into the relationship between LIN28B and miRNAs [49,50].

Several studies provide evidence that LIN28B represents a clinically significant subgroup in NSCLC. In 2018, the work of Meder et al. confirmed that LIN28B expression is associated with shortened overall survival in patients with lung adenocarcinoma. For experiments, an autochthonous *KRAS*-driven mouse model was used. Immunohistochemical staining of mouse lung tumor tissue sections revealed an increased expression of mesenchymal markers like Slug, Snail and Vimentin upon LIN28B expression, whereas expression of the epithelial marker E-cadherin was decreased. These results indicate that LIN28B potentially plays a significant role especially in the process of EMT in *KRAS*-driven lung adenocarcinomas [51].

Moreover, Sato et al. demonstrated that LIN28B also played a role in acquired resistance to EGFR-tyrosine kinase inhibitors (TKI) in NSCLC. Initial qPCR and database analyses revealed that the miR-200 family members, which are known to be negatively involved in EMT, were frequently repressed in several different NSCLC cell lines, and that this silencing was positively correlated with LIN28B overexpression and promoted EMT. Subsequently, ectopic expression of miR-200c, specifically, and the induced knockdown of LIN28B in acquired EGFR-TKI resistant NSCLC cells resulted in an anti-tumorigenic effect and attenuated EMT features. To summarize, these findings identified the miR-200c/LIN28B axis as an important driver in the acquisition of therapy resistance to EGFR inhibition and declared it as an interesting useful therapeutic target [14].

miR-200 along with let-7 miRNA have been linked to cancer stemness, EMT and TKI-therapy resistance in NSCLC by the sonic hedgehog signaling pathway [52]. So far, only developmental processes have been linked to LIN28B and sonic hedgehog signaling [53]. Interestingly, medulloblastomas of the brain frequently harbor mutations in sonic hedgehog signaling and they have shown sensitivity to Aurora kinase inhibitors, inducing cell-cycle arrest and enhancing sensitivity to chemotherapy [54]. It remains to be tested, whether LIN28B can be mechanistically linked to sonic hedgehog signaling especially under TKI-resistance. Related to this, a currently recruiting clinical trial (NCT05017025) combines the Aurora Kinase inhibitor LY3295668 with the TKI-inhibitor Osimertinib in advanced or metastatic EGFR-mutated non-squamous NSCLC.

It is possible that LIN28B contributes to TKI-resistance in NSCLC via Aurora kinase-mediated signaling, and that Aurora kinase inhibition may improve sensitivity to TKI-based treatment.

Altogether, these findings further support the critical oncogenic properties of LIN28B in tumors of the respiratory tract. Furthermore, it has been disclosed that miRNAs, as key up- and downstream regulators of LIN28B, can be important players in tumor progression and therapy resistance.

### Gastrointestinal and urinary tract

The gastrointestinal tract comprises of the mouth, pharynx, esophagus, stomach, small intestine and colon, rectum and anus. Additionally, it includes the salivary glands, liver, gall bladder and the pancreas [55]. In 2018, the number of new cases of gastrointestinal cancers was estimated to be 4.8 million worldwide, and the number of related deaths was indicated with 3.4 million cases. In total, gastrointestinal cancers account for 26% of all newly diagnosed cancer cases and for 35% of all cancer-related deaths [56]. Additionally, numerous studies in these cancer entities support the important role and effects of LIN28B in the context with tumor growth and metastasis formation.

The work of Hamano et al. in 2012 confirmed the upregulation of LIN28B in cancer tissues from patients with esophageal cancer compared to non-cancerous tissues. Moreover, clinical analyses showed that esophageal cancer patients with high LIN28B expression had shorter overall and disease-free survival, as well as lymph node metastasis. Lymphatic vessel invasion was additionally shown to be correlated with LIN28B expression. Further *in vitro* assays confirmed the initial clinical results that LIN28B was associated with tumor aggressiveness and metastasis formation. MTT cell proliferation and Matrigel-based cell invasion assays of LIN28B-knockdown TE-13 and TE-10 esophageal cancer cell lines demonstrated that their proliferative activity and invasion ability were significantly reduced compared to control cells with normal LIN28B expression [57]. Besides, a positive correlation between high LIN28B expression and the proliferation, migration and invasion of gastric cancer cells could also be elucidated. One study also provided that the activity of LIN28B could be affected by the epigenetic methylation pattern of the *LIN28B* gene, namely that *p* hypomethylation positively influences LIN28B expression [58,59].

In hepatocellular carcinoma (HCC), Zhang et al. discovered that the long non-coding RNA, LIN28B-AS1, regulating the activity of LIN28B as demonstrated in the already described work of Wang et al. [47], is expressed in HCC cells and primary HCC tissues. It is known that IGF2BP1 is a direct target of LIN28B-AS1, which was here confirmed with an RNA-immunoprecipitation in HCC. Additionally, *in vitro* and *in vivo* xenograft experiments revealed that LIN28B-AS1 silencing repressed IGF2BP1-dependent mRNAs, and thus inhibiting tumor progression. These findings illustrated that long non-coding RNAs that regulate LIN28B and other mRNAs can influence tumor growth indirectly [60]. In the context of the interplay of miRNAs and LIN28B, another publication found that the miR-125a with antiproliferative activity was downregulated in HCC and was a direct target of LIN28B. LIN28B overexpression reduced the abundance of miR-125a, significantly diminishing its anti-proliferative effects, leading to an increased cell proliferation in HCC [61]. Regarding therapy resistance, Tian and colleagues found that LIN28B was upregulated in paclitaxel-resistant HCC cells. LIN28B was able to reduce the paclitaxel-induced apoptosis of the cells. Additionally, they could show that Curcumin could attenuate the paclitaxel-induced chemoresistance by inhibiting NF-κB induced LIN28B expression [18]. Subsidiary, two further publications also indicated of the positive association of LIN28B expression and cell transformation and tumor progression in HCC [62,63]. Regarding cholangiocarcinoma (CCA), Yang et al. established a chemically induced mouse model leading to CCA progression in 2011. Western Blot assays revealed that LIN28B expression was induced during later stages of cholangiocarcinogenesis, together with let-7a downregulation. Correspondingly, miR-34a was downregulated while miR-210 was upregulated. Because LIN28B expression was highest in the advanced stages of CCA, they concluded that LIN28B plays an important role in hyperplasia and lesion formation in CCA [64].

Another study disclosed a relation between LIN28B and TGF-β signaling in CCA. Several *in vitro* experiments showed that LIN28B overexpression promoted cell migration, invasion and the expression of EMT markers in cholangiocyte and CCA cell lines. Additionally, they found an upregulation of the transforming growth factor beta induced protein (TGFBI), the TGF-β receptor 1 (TGFBRI) and an elevated secretion of TGF-β1, -β2 and -β3 upon LIN28B overexpression. Thus, a positive LIN28B/TGF-β/TGFBI feedback loop was created and CCA aggression was promoted [65]. Besides, Puthdee et al. reported in 2021 that LIN28B could induce chemotherapeutic resistance via enhancing STAT3 signaling in cholangiocytes [17]. TGF-β signaling has also been identified as an important molecular pathway in pancreatic ductal adenocarcinoma (PDAC). TGF-β treatment induced the expression of the long non-coding RNA MIR100HG, containing miR-100, miR-125b and let-7a in various PDAC cell lines. While pro-tumorigenic miR-100 and miR-125b levels increased, anti-tumorigenic let-7a levels remained unchanged. According to RNA-sequencing data, TGF-β simultaneously stimulated LIN28B expression, which in turn inhibited let-7a levels. Induced knockdown of both miR-100 and miR-125b strongly impaired the EMT and stemness properties of the PDAC cell lines. These expression correlations could be confirmed in PDAC patient samples as well. This means that TGF-β simultaneously induced the expression of miR-100, miR-125b, let-7a, and LIN28B expression. MiR-100 and miR-125b could carry out their pro-tumorigenic effects, whereas increased LIN28B expression inhibited the anti-tumorigenic let-7a, altogether resulting in promoted EMT and stemness of the PDAC cells [66]. Next, the work of Franses and colleagues provided a potential promising therapeutic strategy for metastasis prevention. With the CTC-iChip technique, they characterized purified circulating tumor cells (CTCs) from PDAC patients with RNA-sequencing and found three major enriched clusters of stemness genes: LIN28B/KLF4, WNT5A and LGALS3. They demonstrated that this diagnostic technique could potentially serve as a “liquid biopsy” to identify and generate potential biomarkers of response to therapeutic drugs, such as LIN28B, and thus to prevent CTC abundance and metastasis formation by confirming the LIN28B knockout attenuated PDAC aggressiveness *in vitro* and *in vivo* [67]. Furthermore, Zhang et al. showed that pancreatic cancer cell-derived exosomes aided in the recruitment of pancreatic stellate cells, specifically the cancer-associated fibroblasts (CAFs) of the tumor microenvironment (TME). In the recipient cells, the exosomal LIN28B activated the LIN28B/let-7/HMGA2/PDGFB signaling pathway and thus promoted cell recruitment [68]. The role of LIN28B in epigenetics could be underlined by the publication of Kugel and colleagues. Here, Sirtuin 6 (SIRT6), a histone deacetylase, normally suppresses LIN28B expression and thus PDAC progression. When SIRT6 is not expressed, histone hyperacetylation at the LIN28B promoter is induced, its expression is stimulated, and its downstream let-7 target genes, such as HMGA2, IGF2BP1, and IGF2BP3 are upregulated [69]. Last but not least, Liu et al. demonstrated that LIN28B was upregulated in pancreatic cancer and contributed to cell proliferation and migration. They found that KRAS promoted the nuclear translocation of LIN28B and that the KRAS/Lin28B axis drove the let-7i/TET3 pathway to maintain the stem-cell like phenotype of pancreatic cancer cells [70].

The publication from Tu and coworkers in 2015 identified LIN28 as a crucial component for tumor progression and maintenance in colorectal cancer as well. They discovered that both LIN28A and LIN28B expression promote tumor initiation, but LIN28B-induced tumors more frequently progressed to advanced adenocarcinomas in intestinal tissues and in a genetic mouse model. This suggested that LIN28B was crucial for invasive aggressiveness and tumor maintenance. They also revealed a nuclear/cytoplasmic β-Catenin translocation and an elevated expression of Wnt target genes in LIN28A and LIN28B induced tumors. A next-generation deep sequencing analysis demonstrated that several tumors in this approach also harbored somatic mutations in the *Cnnb1* gene. Thus, they concluded that LIN28A and LIN28B cooperated with the activated Wnt signaling to promote intestinal tumorigenesis [71]. Additionally, the studies of Tang et al. and Wang et al. both investigated the interplay of miRNAs and LIN28B and the effects of cancer progression in colorectal cancer (CRC) [72,73]. Several more publications provide further insight regarding LIN28B’s stimulating effect on cancer progression, metastasis formation, and therapy resistance in the colon cancer entity [74–79].

In case of the urinary tract, which is comprised of the kidneys, the ureter, the bladder and the urethra [80], two publications illuminate the pro-tumorigenic role of LIN28B until now. The publication of Urbach et al. showed that both LIN28 paralogs were overexpressed during kidney development in mice and significantly blocked differentiation, eventually leading to the formation of Wilms tumor. LIN28 inhibition impaired tumorigenesis and enforced expression of the let-7 miRNA. In addition, LIN28B overexpression could be specifically determined in a significant percentage in Wilms tumor. Thus, they identified the Lin28/let-7 pathway as a crucial component in kidney development and tumorigenesis [81]. In bladder cancer, the long non-coding RNA LINC01451 was found to bind directly LIN28A and LIN28B and thus provoked the proliferation, invasion, and metastasis of bladder cancer. Further investigation identified the activated TGF-β/Smad signaling pathway promoting EMT and bladder cancer progression [82].

Collectively, these publications further enforce the contribution of LIN28B activity in tumor initiation, progression and metastasis formation in several different organs of the gastrointestinal and urinary tract.

**Figure 3:**
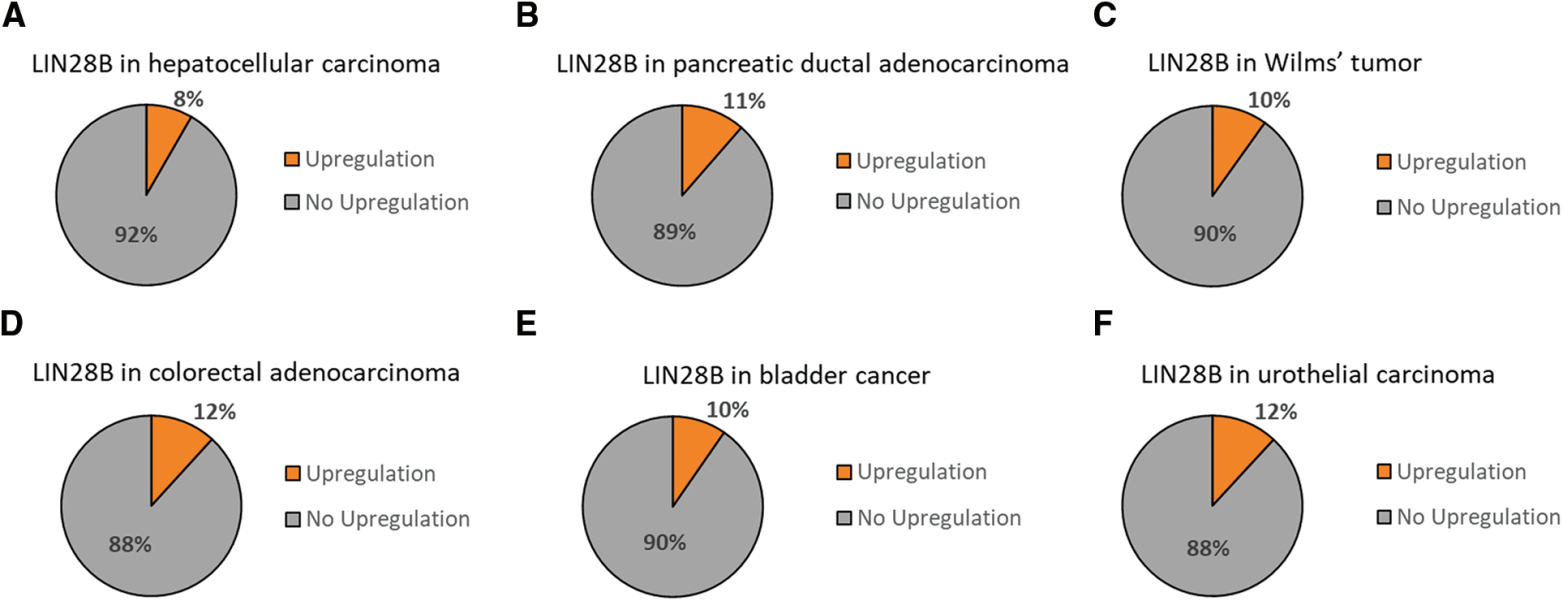
LIN28B upregulation in solid tumor entities of the gastrointestinal and urinary tract. TCGA data sets for hepatocellular carcinoma [83], pancreatic ductal adenocarcinoma [84], Wilms tumor [85], colorectal adenocarcinoma [86], bladder cancer [87] and urothelial carcinoma [88] have been analyzed for LIN28B upregulated fractions in cBioportal [7,8].

Moreover, TCGA data analysis using cBioportal [7,8] revealed that LIN28B marks a clinically relevant subgroup in these cancer entities [83–88] (Fig. 3). Finally, the promising potential usage of LIN28B as a diagnostic tool and therapeutic target could be illustrated again.

### Reproductive system

**Figure 4:**
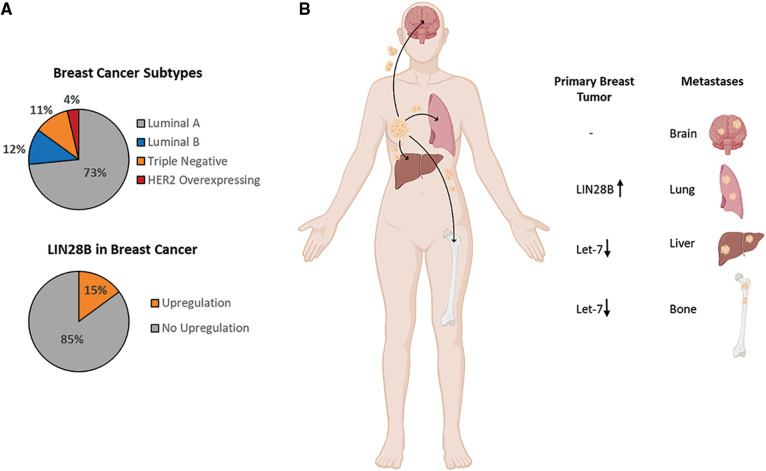
LIN28B in Breast Cancer and Breast Cancer Metastasis. (A) Breast cancer subtype distribution among the whole breast cancer entity [91], and the LIN28B upregulation, including amplification and high mRNA expression, in a breast cancer cohort of 463 patients (TCGA, Nature 2012, [92]) [7,8]. (B) Illustration of the most common metastatic sites in breast cancer patients and the LIN28B or let-7 feature in the primary breast tumor contributing to metastasis formation at specific sites [89,90,94–96]. Created with BioRender.com.

With over 268,000 new diagnosed cases per year, breast cancer is the most diagnosed malignancy among women in the USA and represents the second most cause of cancer-related death. In addition, it shows a high metastatic heterogeneity which implies multiple distant metastatic sites, including bone, liver, lung and brain. With a 5-year overall survival rate of only 22.8%, bone metastasis seems to be the most common metastatic destination, necessitating further research for more effective therapy or molecular biomarkers for diagnosis [89,90]. Generally, breast cancer can be divided in four major subtypes: Luminal A, Luminal B, triple negative, and HER2-positive. The percentage of them in the overall breast cancer entity is depicted in Fig. 4A [91]. According to an analysis of a breast cancer cohort (463 cases, the Cancer Genome Atlas (TCGA), Nature 2012 [92]), LIN28B is upregulated in 15% of breast cancer patients (Fig. 4A) [7,8]. Especially in the triple negative subtype, the upregulation of LIN28B seems to be the most prominent among the other subtypes [93,94]. Furthermore, functional *in vivo* experiments from different studies revealed that LIN28B overexpression and let-7 downregulation in primary breast tumors of mouse models are directly linked to distant metastasis formation as illustrated in Fig. 4B [94–96]. Unfortunately, no studies were published yet regarding the brain metastasis formation in breast cancer.

Interestingly, low pH in breast cancer caused by high levels of lactate increased cancer stemness as represented by LIN28B, Myc, and aldehyde dehydrogenase 1 (ALDH1) expression. In addition, miR34a was downregulated by LIN28B to trigger glycolysis instead of oxidative phosphorylation [97]. MiRNA-34 and miRNA-200 counter regulate the EMT-transcription factors, Snail and ZEB1, and thereby contribute to partial EMT, a critical feature of metastatic processes [98,99]. In this regard, the publication of Dupuy et al. is striking, as they highlighted that breast cancer cell metabolism dictates the side of metastasis. Tumor cells favoring glycolysis invade the liver, whereas tumor cells favoring oxidative phosphorylation migrate to the lung and the bone [100]. It remains elusive whether LIN28B expression can favor a certain site of metastasis, since it has been previously functionally linked to lung and liver metastasis of breast cancer (Fig. 4).

A further important aspect in tumor progression and metastasis is the modulation of the immune micromilieu. The study of Qi et al. showed that the pluripotency factor LIN28B also executes its tumor promotive effects in breast cancer and additionally can promote the formation of pre-metastatic niches at metastatic sites *in vitro*, *in vivo* and in human breast cancer patients. Initially, clinical analysis of breast cancer patients’ data revealed higher LIN28B expression in tumor tissues, as well as a positive correlation between LIN28B and clinical tumor grade, distal lung metastases, and shorter overall post-surgery survival. To inspect the accurate role of LIN28B in metastasis formation, an orthotopic mouse model with LIN28B-transduced murine breast-cancer 4TO7 cells was used. Tumor resection exposed a stimulatory effect of LIN28B expression on the incidence of lung metastases. Furthermore, neutrophils were especially enriched in the pre-metastatic lung niche upon LIN28B expression. LIN28B stimulated the N2 conversion of the accumulated neutrophils by upregulating IL-6 and IL-10 expression in the metastatic site. Additionally, the N2 neutrophils were able to create an immunosuppressive milieu in the pre-metastatic lung by upregulating PD-L2 on their surface. Moreover, to explore the detailed role of tumor-derived exosomes, a qPCR analysis exposed a lower content of the let-7 family members in the LIN28B-exosomes compared to the controls. Further experiments showed that a lower amount of the let-7 family members in the tumor-derived exosomes was the major driver for the immunosuppression in the pre-metastatic lung. In summary, Qi and coworkers here identified a new mechanism of LIN28B promoting breast cancer metastasis formation, which includes the creation of an immunosuppressive microenvironment in the secondary tumor site via tumor-delivered exosomes [94]. Another study found that LIN28B is involved in inflammatory tumor responses as a result of epigenetic switches in tumor cells. Here, a tamoxifen-inducible experimental setup with the immortalized breast cancer cell line MCF10A was used. The treatment led to a phenotypic transformation with a more invasive character and an increased mammosphere formation ability. Different quantification assays revealed that upon tamoxifen treatment, Src stimulated the activity of the NF-κB transcription factor. This results in, in addition to an increased IL-6 dominated inflammatory response, activation of LIN28B. Subsequently, important oncogenic molecules like Ras and the STAT3 transcription factor were upregulated via the suppression of let-7, eventually transforming the cells to be pro-tumorigenic [101]. A further study wherein the previous author was also involved, demonstrated that LIN28B overexpression could diminish the inhibiting effects of metformin on the breast cancer cell transformation, and thus can contribute to therapy resistance [19].

**Figure 5:**
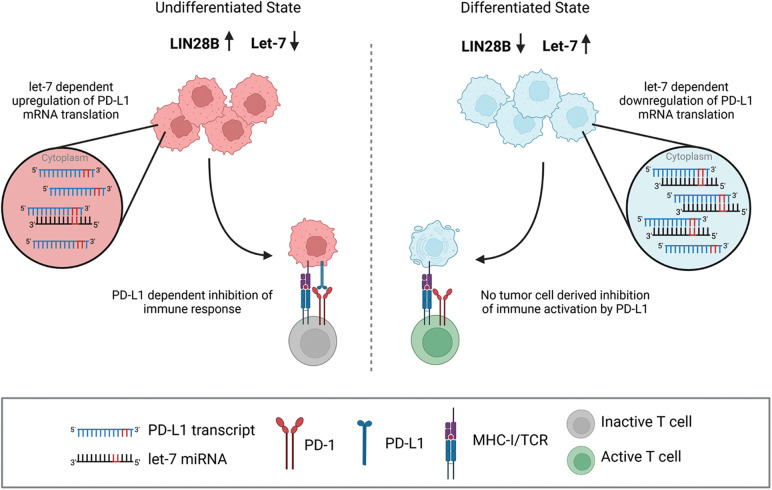
LIN28B can trigger escape from immune surveillance. The LIN28B target let-7 has three putative binding sites on the PD-L1 mRNA and represses PD-L1 expression on tumor cells [102]. MHC-I/TCR signaling triggers T cell activation. Once PD-L1 is upregulated in LIN28B expressing tumor cells, it may interact with its PD-1 receptor and thereby inhibit an anti-tumor T cell response. Created with BioRender.com.

The study of Chen et al. additionally showed that the LIN28/let-7 pathway was involved in the immune evasion of breast cancer cells amongst others, which lead to therapy resistance to programmed death receptor-1 (PD-1)/programmed death-receptor-ligand-1 (PD-L1) immune checkpoint therapeutics. They identified an inverse correlation of let-7a miRNA and PD-L1 expression in a cohort of TCGA breast cancer samples and found three putative target sites for let-7 on the PD-L1 mRNA. Ultimately, they confirmed that let-7 repressed PD-L1 expression, and that when LIN28B was upregulated in cancer cells, PD-L1 expression was indirectly increased, promoting immune evasion or therapy resistance to PD-1/PD-L1 immune checkpoint inhibitors *in vitro* and *in vivo* [102]. In summary, LIN28B is an immunomodulatory factor that contributes to tumor escape from immune surveillance (Fig. 5).

Next, more publications, including the work of Ji et al., demonstrated that long noncoding RNAs can function as miRNAs sponges, indirectly leading to the upregulation of LIN28B. This would then promote cancer progression, further strengthening the important oncogenic role of LIN28B in breast cancer [97,103–106]. Another type of women’s cancer is the ovarian cancer, which has a global incidence rate of 225,500 new cases per year and also a very poor prognosis for patients, particularly those in the late stages of disease [107]. Lin et al. could show that there was also a link between LIN28B expression and reduced apoptosis in ovarian cancer cells. After initially demonstrating that LIN28B high expression in patients with ovarian cancer significantly reduced overall survival, following experiments revealed that the knockdown of LIN28B significantly increased the number of apoptotic cells. Additionally, the protein levels and activity of cleaved caspases, which are crucial mediators in the apoptotic pathway, were significantly upregulated. According to this, LIN28B overexpression showed the opposite effects. To find the molecular mechanism behind this anti-apoptotic effect, they investigated the expression changes in key regulators of the apoptosis pathway dependent on LIN28B expression. They could identify that LIN28B functions via the AKT2/FOXO3A/BIM axis, finally leading to an inhibited expression of the pro-apoptotic BIM protein and thus to an increased resistance to apoptosis, when LIN28B is overexpressed [108]. Furthermore, the work of Hsu et al. on the one hand reported a correlation between LIN28B expression and increased proliferation, migration and invasion in ovarian cancer, while on the other hand revealing that LIN28B increased the resistance to the chemotherapeutic agent Cisplatin [109]. Additional studies further support the association between high LIN28B expression and advanced clinical tumor stage, worse survival rates and increased cell proliferation and migration in this tumor entity [110–112]. Also in endometrial squamous carcinoma, a subtype of uterine cancers, a study once more indicated the important role of miRNAs in this context [113]. Adding on, LIN28B exerts its pro-tumorigenic effects in prostate cancer with 1,414,000 new cases estimated worldwide in 2020 [114]. Lovnicki and coworkers showed that LIN28B played a crucial role in tumorigenesis in the therapy-induced neuroendocrine prostate cancer (t-NEPC) subtype. This highly aggressive subtype emerges upon anti-androgen receptor therapy as the adenocarcinoma cells undergo redifferentiation and transform into cells with neuroendocrine phenotype, finally turning into t-NEPC. Here, they performed different *in vitro* and *in vivo* experiments, including LIN28B overexpression and LIN28B knockout via CRISPR approaches. The results disclosed the molecular pathway over which LIN28B carried out its pro-tumorigenic properties. LIN28B was upregulated during redifferentiation, leading to the suppression of the let-7 miRNA and, finally, the HMGA2-mediated upregulation of SOX2, resulting in the formation of a cancer stem-cell-like phenotype and the development of t-NEPC. This work clearly depicted LIN28B as a key initiator of a redifferentiation cascade, explaining the more aggressive growth pattern of prostate cancer cells. Such findings further pronounce the promising potential application of LIN28B as a molecular therapeutic target and biomarker [115]. Moreover, the important role of miRNAs in tumor progression has been reported in this tumor entity. Recently, in 2021 a study demonstrated that the re-expressed miR-26a directly targeted and suppressed the LIN28B protein and the uridyltransferase Zcchc11 in prostate, melanoma and liver cancer *in vitro* and *in vivo*. This led to an upregulation of the let-7 miRNA, which then decreased tumor growth and metastasis formation [116].

In addition, the work of Albino et al. revealed that under normal conditions, the ETS transcription factor ESE3/EHF silenced the promoters of the LIN28A and LIN28B genes. On the other hand, ESE3/EHF was repressed in cancer cells, which led to a rise of LIN28B protein levels and was crucial for cell transformation and expansion of prostate cancer stem cells [117]. Kong et al. and coworkers could show a positive correlation between the expression levels of Oct4, Nanog, LIN28B and Notch1 and the EMT phenotype of prostate cancer cells. Also, the participation of miR-200 and let-7 as negative regulators of EMT were indicated [118].

In summary, these findings provide novel and interesting capabilities of LIN28B regulating the micromilieu of pre-metastatic niches, therapy resistance, cancer cell reprogramming and consequences on tumor progression. It remains elusive till today, whether the immune modulatory effects of LIN28B in the tumor micromilieu affect therapeutic outcome under immune checkpoint blockade, which has been approved for metastatic triple negative breasr cancer (TNBC) [119] and metastatic prostate cancer [120].

### Skin and bone

Skin cancer is normally divided into the malignant melanoma and non-melanoma skin cancer. Malignant melanoma, in particular, accounts for only 4% of all skin cancer cases, however accounts for up to 65% of all skin-cancer related deaths, making it a highly insidious tumor entity [121].

Li and colleagues investigated the mechanism by which miRNAs control metastasis in melanoma. A correlation between decreased miR-98 expression in melanoma tissues and increasing tumor stage, metastasis occurrence, and patient survival was found using patient data. Different *in vitro* and *in vivo* experiments revealed miR-98 inhibited metastasis in melanoma cells. Mechanistically, *IL-6* was identified as a target gene of miR-98, and IL-6 repressed miR-98 levels via STAT3/NF-κB/LIN28B pathway creating a negative feedback loop. Thus, when miR-98 was decreased, IL-6 and LIN28B were elevated and contributed to melanoma progression [122]. Moreover, it has been found that LIN28B is essential for melanoma progression, since it drives a TGF-β signaling cascade in a 7-dependent manner [123].

Next, the work from Zhu et al. also investigated the connection of LIN28B with miRNA metabolism. The data provided that the expression of the miR-10a-5p was significantly decreased in melanoma tissues. Induced overexpression of miR-10a-5p markedly suppressed tumor proliferation, migration and invasion in A375 and B16-F10 melanoma cells. Ultimately, they found that the TCF21/miR-10a-5p/LIN28B axis was at least partially responsible for melanoma progression [124]. Finally, LIN28B has been associated to resistance to X-ray irradiation [125] as well as chemoresistance [126].

In osteosarcoma, the study of Mizushima et al. showed that upon LIN28B inhibition, tumorigenesis and chemoresistance was decreased in OS13 cells and reversed oxidative phosphorylation function [127]. This finding is in concordance with the results of LIN28B-dependent metabolic reprogramming observed in breast cancer, which affected the selection of metastatic sites [89,90]. An applied combination therapy comprised of a glycolysis inhibitor and low-dose chemotherapy resulted in a repression of the tumor. Collectively, the therapeutic regulation of glycolysis together with chemo-therapy might be a good therapy opportunity for osteosarcoma [127].

Keskin et al. go a step further, classifying LIN28B expressing Ewing sarcomas as their own subclass, claiming that LIN28B regulates the stability of the oncogenic EWS-FLI1 fusion protein. As effective therapeutic strategy, they used pharmacological inhibition of LIN28B with the 1632 inhibitor [128].

Overall, these studies show evidence that LIN28B has pro-tumorigenic effects and likely represents a therapeutically relevant subgroup in skin and bone cancer entities.

## Conclusion

The majority of the publications presented here indicate the potential relevance of LIN28B in research as a biomarker for cancer invasion and aggressiveness and in the clinic as a therapeutic target and diagnostic tool. Unfortunately, no studies have been published that directly use LIN28B occurrence as a biomarker for tumor grading or evidence of metastasis. However, a strong positive correlation of LIN28B expression and shortened overall survival, advanced clinical stages and metastatic burden was shown in different tumor entities [25,38,40,51,57,74]. These correlation patterns are already used in preclinical research for the generation of invasive tumor cell lines or genetic cancer mouse models to investigate invasive and metastatic tumor stages of a desired tumor entity.

The determination of LIN28B levels in tumor biopsies from patients, for example, could be used to get an idea of tumor stage and invasiveness. The previously described study by Franses and colleagues, for instance, applied the CTC-iChip technique to purify circulating tumor cells from the blood of PDAC patients, resulting in a “liquid biopsy,” and characterized them via RNA-sequencing, including LIN28B, displaying a very promising diagnostic method for the future [67]. Furthermore, due to LIN28B’s serious pro-tumorigenic effects, it would be a promising therapeutic target in the clinic.

The majority of the LIN28 inhibitors block both isoforms LIN28A and LIN28B due to their structural similarity and act by the disruption of the binding between the ZKD domain of LIN28 and the miRNAs [129]. Currently available LIN28A and LIN28B inhibitors have been extensively reviewed by Lin and colleagues [130]. A few other promising molecules have been developed, including C1632 with its chemically name *N*-Methyl-*N*-[3-(3-methyl-1,2,4-triazolo[4,3-*b*]pyridazin-6-yl)phenyl]acetamide [131]. C1632 has been already successfully used in cancer mouse models to significantly reduce tumor growth [97,102]. Till today, it remains elusive whether this compound has a tolerable safety profile and can be combined with chemotherapy or immune checkpoint blockade in an immunocompetent setting.

Collectively, this review provides a versatile overview of the oncogenic effects of LIN28B in a large variety of solid tumor entities. LIN28B expression significantly promotes tumor growth, tumor progression, cancer cell stemness, cancer aggressiveness and metastasis formation. Until now, the majority of publications published in this field have been represented by the entities brain, pancreas, and breast cancer. Furthermore, several studies represented in this review demonstrate that LIN28B is largely involved in the metabolism of miRNAs and can influence tumor growth properties via miRNA regulation. Finally, LIN28B was also shown to be involved in therapy resistance, particularly TKI-resistance in lung cancer.

Altogether, the expression pattern of LIN28B in tumor cells, as well as its highly promoting effects on tumor progression and metastasis formation, make it a highly promising therapeutic target, and it may also serve as a biomarker supporting the diagnosis regarding tumor stage and aggressiveness.
